# Encapsulation improves viability and stability of spray-dried *Lactococcus lactis* A12 for inclusion in fish feed

**DOI:** 10.1371/journal.pone.0323000

**Published:** 2025-05-27

**Authors:** Marcelo Fernando Valle Vargas, Ruth Yolanda Ruiz Pardo, Luisa Villamil-Díaz, Jader Alean, Patricio Román Santagapita, María Ximena Quintanilla-Carvajal

**Affiliations:** 1 Grupo de Investigación en Procesos Agroindustriales (GIPA), Doctorado en Biociencias, Facultad de Ingeniería, Universidad de La Sabana. Campus del Puente del Común, Km. 7, Autopista Norte de Bogotá, Chía, Cundinamarca, Colombia; 2 Universidad de La Guajira, Facultad de Ingeniería, Riohacha, La Guajira, Colombia; 3 Universidad de Buenos Aires, Facultad de Ciencias Exactas y Naturales, Departamento de Química Orgánica, & Centro de Investigación en Hidratos de Carbono (CIHIDECAR, UBA-CONICET), Buenos Aires, Argentina.; Universidad Autonoma de Chihuahua, MEXICO

## Abstract

During probiotics manufacturing, drying is a crucial process for stabilization of probiotics after fermentation, since drying condition could affect viability and functionality as well as physical properties such as moisture content and water activity, which play key role in stability of dried probiotics during storage. Therefore, this study aimed to evaluate the effect of spray-drying parameters on the survival of *Lactococcus lactis* A12 after drying and exposure to gastrointestinal conditions. A combined mixture-process design was carried out by evaluating three factors: whey (10–30% w/v), maltodextrin (10–30% w/v), and atomization pressure (1.0–1.5 bar). As the main results, a high concentration of whey (30% w/v), low concentration of maltodextrin (10% w/v), and high atomization pressure (1.4 bar) improved survival of spray-dried *L. lactis* A12 after drying and exposure to pH 3.00 or bile salts with survival rates ranged within 69.25 to 86.24%, 65.89–98.93%, and 89.09–100%, respectively. Under optimal conditions, spray-dried probiotic powder with wall materials (encapsulated) exhibited higher glass transition temperature (64.44 vs 12.65 °C), and lower hygroscopicity (12.65 vs 64.44%) than spray-dried probiotic without wall materials (non-encapsulated). Moreover, SD probiotic powder exhibited the highest survival rate (85.88%) at 4 °C during 60 days of storage in comparison to 25 °C and 37 °C which did not survive. Finally, spray-dried *L. lactis* A12 was included in fish feed and exhibited a survival rate of 80.83% when it was stored at 4 °C after 60 days. It can be concluded that the use of encapsulating materials, particularly whey and maltodextrin, improved the physical and thermal stability of *L. lactis* A12 powder during drying and storage. Also, the results from the stability of supplemented fish feed suggested that *L. lactis* A12 could be included in fish feed.

## 1. Introduction

According to the estimation of the Food and Agriculture Organization (FAO), by 2050 global food production must increase by 60% to feed 9.3 billion people. Aquaculture stands as a crucial sector, providing fishery products as a key protein source for human consumption [1]. In 2022, global production of aquatic animals reached an estimated 185 million tons, with capture fisheries and aquaculture contributing 49% and 51% of the total output, respectively. Within aquaculture production, (94 million tons), inland aquaculture accounted for 84%, with Tilapia and Carp being the major harvested species [2]. The rising demand for aquatic food, particularly fish species, has led to intensified fish farming practices characterized by high stocking densities, deteriorating water quality, and the emergence of anthropogenic stressors, all of which can render fish susceptible to infections and diminish growth performance [3–5]. In response to managing these infections, fish farming producers have turned to antibiotics for treating bacterial diseases increasing the emergence of antimicrobial-resistant microorganisms [6].

Probiotics are an efficient and environmental approach to reduce the use of antibiotics and mitigate the propagation of antimicrobial resistance [7]. Probiotics are microorganisms that when ingested in adequate amounts confer health benefits to the host [8]. Administration of probiotics to animals, especially fish, improve the immune system, microbiota, resistance against pathogens, feed conversion, nutrient digestibility, weight gain, body composition, and quality of fish [9–11]. Bacteria from genera *Bacillus*, *Lactobacillus*, and *Lactococcus* have been studied for their probiotic potential in fish species [12]. Among these microorganisms, *L. lactis* is of great interest since some strains produce bacteriocins [13,14] that have shown *in vitro* [15] and *in vivo* probiotic potential in fish [16,17]. *Bacillus* have been widely studied as probiotics in aquaculture for their beneficial characteristics such as survival under gastrointestinal conditions and high temperatures [18]. In addition, some *Bacillus* strains have exhibited *in vitro* [19,20] and *in vivo* probiotic potential in Nile tilapia [21–23]. Nevertheless, probiotics could face several challenges from their production in bioreactors, drying, storage, and inclusion in fish feed, until their ingestion and passing through the gastrointestinal tract of the fish [24–26]. In the gastrointestinal tract, probiotics face harsh conditions; in the stomach, the presence of acid enzymes such as pepsin, mostly, and low pH affect probiotic viability, in the intestine, enzymes such as trypsin and chymotrypsin as well as bile salts affect cell integrity causing loss of viability [27]. Therefore, probiotic protection strategies such as encapsulation should be used to protect the probiotic from external factors during production, storage, and passing through the GIT tract.

Encapsulation consists in the entrapment of an active compound (core, probiotic bacteria) inside a matrix (wall materials) [28]. Probiotics have been encapsulated by different techniques, spray-drying and freeze-drying are the most used to produce dried powders [29]. On one hand, spray-drying is a well-established technique due to its high efficiency and low cost [30], however, its main disadvantage is the use of high temperatures that could affect bacteria viability [24]. On the other hand, freeze-drying is a technique that uses low temperature and high vacuum conditions to obtain probiotic powders with high viable cell counts [31]. Its main disadvantage is the high energy consumption of 10 MJ/kg of evaporated water compared to spray-drying (6 MJ/kg of evaporated water) [32] and the energy-related costs that could be four times higher than spray-drying [33].

Although spray-drying causes bacterial loss due to high temperature, addition of wall materials could improve bacterial survival after the process [34] and increase the product stability during storage [35]. Polymers such as maltodextrin, starch, chitosan, alginate, whey, milk proteins, sodium caseinate, gelatin, among others, and lipids have been used for encapsulation of probiotics by spray-drying [36]. Among these wall materials, whey has been used for the protection of probiotics during drying [37] and *in vitro* digestion [38].

Several factors could affect the survival of probiotic bacteria during spray-drying and exposure to GIT conditions including the wall material, inlet and outlet temperature, air pressure atomization, feed rate, etc [39–41]. Studies have reported the encapsulation of lactic acid bacteria by spray drying including *Lactobacillus* [34,35,42] and *Lactococcus* species [43–47], however, few studies have focused on evaluating the effect of the process parameters on the survival of probiotic bacteria after drying and under exposure to gastrointestinal conditions. Aragón-Rojas et al. [42] evaluated the effect of inlet temperature and atomization pressure on the survival of *L. fermentum* K73 after drying and under gastrointestinal conditions. They found that low outlet temperature (90.8 °C) and air pressure (1.17 bar) lead to low bacterial change cycles after drying (-2.0 ± 0.2 log_10_), exposure to pH 2.00 (-0.61 ± 0.08 log_10_), and bile salts (-0.20 ± 0.00 log_10_).

It has been shown that encapsulated probiotics has improved growth performance parameters compared to non-encapsulated ones during *in vivo* trials in Nile tilapia [26,48]. Previous studies from our research group have reported the *in vitro* potential of a multistrain probiotic composed of *L. lactis* A12, *Priestia megaterium* M4, and *Priestia* sp. M10 [49]. These bacteria were produced in commercial medium (BHI), mixed with fish feed, and freeze-dried [50]. Recently, an agro-industrial by-products based medium composed of whey, sugarcane molasses, and palm kernel cake was designed to produce these probiotic bacteria in monoculture [51] and coculture [52] conditions. Finally, these probiotic bacteria were produced in bioreactor using the designed culture media and exhibited probiotic properties such as tolerance to low pH and bile salts, and antibacterial activity against pathogen *S. agalactiae* [53]. The next step is to evaluate if this culture media could be used as a double purpose media for both biomass production and dissolving medium for encapsulating materials. Previous studies have reported the use of whey as double use media for biomass production of *Lactobacillus* and encapsulation by spray-drying [42,54]. These approach avoid the centrifugation of culture media and washing of bacterial cell pellets for the drying process [55].

Even though the use of double purpose culture has been reported, the use of this approach for production and encapsulation of probiotic bacteria intended for animal nutrition, especially fish species, has not been reported in literature. Moreover, the lack of studies focused on the influence of process parameters on the survival of probiotics, especially for *L. lactis* strains after drying and exposure to gastrointestinal conditions, highlights the necessity for more detailed research. Therefore, this research aimed to evaluate the effect of wall materials mixture and atomization pressure on the viability of *L. lactis* A12 after spray drying as an encapsulation technique and exposure to gastrointestinal conditions.

## 2. Materials and methods

### 2.1. Materials

Sodium casenaite (Ciacomeq S.A.S., Colombia), gum arabic (Ciacomeq S.A.S., Colombia), high methoxy pectin (degree of esterification of 70%, Ciacomeq S.A.S., Colombia), maltodextrin (DE 20–23, Grain Processing Corporation, USA), sweet whey (Saputo, USA), sodium alginate (M/G ratio: 0.9 and MW 14 kDa, Sigma Aldrich, USA), gelatine type A (100 kDa, Cimpa S.A.S., Colombia), modified starch (Ingredion, USA), and starch (Cimpa S.A.S., Colombia) were used in this study.

### 2.2. Ethical statement

The project followed the Colombian national government’s regulations. The Permit for accessing genetic resources was issued by the Colombian Ministry of Environment Number 117 (Otrosí4) on the 8th of May 2018 for five years.

### 2.3. Microorganisms

*L. lactis* A12, *P. megaterium* M4, and *Priestia* sp. M10 were isolated from a competitive bacterial exclusion culture derived from Nile tilapia (*O. niloticus*) gut microbiota [56]. Potential probiotic bacteria were identified by molecular techniques and sequenced the whole genome in a previous study [49]. Bacteria were deposited under code A12 (*Lactococcus lactis* A12), M4-MR4 (*Priestia megaterium* M4), and M10-MR10 (*Priestia* sp. M10) in the Chilean Collection of Microbial Genetic Resources (CChRGM) at the Instituto de Investigaciones Agropecuarias (INIA, Chillan, Chile). This institute is registered in the World Data Centre for Microorganisms (WDCM) with registration number 1067. These bacteria were stored in 1.5 mL Eppendorf tubes with BHI (Brain Heart Infusion medium Oxoid, UK) and 40% v/v glycerol at -20 °C in a bacterial suspension: BHI volume ratio of 1:1. Bacteria were activated on TSA (Tryptic Soy Agar, Sharlau, Spain) at 28 °C for 48 h. Then, a single colony of each bacteria was taken from the TSA, inoculated in BHI broth, and incubated overnight at 28 °C. This suspension was the inoculum.

### 2.4. Preparation of culture medium and fermentation condition

The methodology described by Valle-Vargas et al. [52] was used. Briefly, a 250 mL shake flask containing 90 mL of BHI broth (previously sterilized) was inoculated with 10 mL of inoculum (61% *L. lactis* A12, 23% *Priestia* sp. M10, and 16% v/v *Priestia megaterium* M4) with a cell concentration of 6.00 log_10_ CFU/mL. Inoculated BHI broth was placed in an orbital incubator shaker (Innova 42, New Brunswick Scientific, USA) at 28 °C and 100 RPM for 7 h. In the meantime, 900 mL of culture medium composed of 1.00% whey powder, 0.50% sugarcane molasses, 0.77% palm kernel cake, 0.84% yeast extract, 2.63% Na_2_HPO_4_, and 94.26% water was prepared and added in a 1.7 L bioreactor. The final mixture was sterilized at 121 °C for 15 min. After that, the culture medium contained in the bioreactor was inoculated with the 7-hour bacteria grown in BHI broth. Then, the bioreactor conditions were set agitation speed (150 RPM), temperature (28 °C), and incubation time (17 h). Finally, after the process was finished, samples of the final culture medium with grown probiotic bacteria were taken to evaluate the final cell concentration (log_10_ CFU/mL) of *L. lactis* A12 and *Priestia* species [52].

The culture composition and agitation speed in the bioreactor were achieved in a previous study through an optimization design [53].

#### 2.4.1. Quantification by High Performance Liquid Chromatography (HPLC) of sugars and organic acids.

The final culture medium with grown bacteria (10 mL) were filtered through a Nylon membrane syringe filter (0.22 µm, 25 mm diameter disk filters, CNW, China). Concentrations of glucose, fructose, lactose, sucrose, xylose, galactose, and lactic and acetic acids were quantified using a HPLC LC-20AT (Shimadzu, Japan) equipped with a refractive index detector (RID-20A).

For the detection of sugars, a column Aminex HPX-87P (300 × 7.8 mm × 9µm) with a precolumn Bio-Rad CarbonP (30 × 4.6 mm) was used. The mobile phase consisted of water at a flow rate of 0.6 mL/min at 80 °C. For the detection of organic acids, a column Aminex HPX-87H (300 × 7.8 mm × 9 µm) with a precolumn Bio-Rad Cation H^+^ (30 × 4.6 mm) was used. The mobile phase consisted of 5mM sulfuric acid at a flow rate of 0.6 mL/min at 65 °C. Standards of the respective sugars and organic acids were used to quantify the compounds.

### 2.5. Selection of wall materials

Nine wall materials were tested individually (high methoxy pectin, gum arabic, maltodextrin, sweet whey, sodium caseinate, sodium alginate, starch, modified starch, and gelatin type A according to the methodology described by Aragón-Rojas et al. [42] with some modifications. 50 mL of culture medium containing growing bacteria (*L. lactis* A12 and *Priestia* species 9.29 ± 0.11 and 6.59 ± 0.16 log_10_ CFU/mL, respectively) from the bioreactor were mixed with solutions ranging from 2 to 40% w/v of each wall material (depending on dissolution of wall materials) in a 250 mL flask and homogenized until completed dissolution. Then, a sample from each mixture was taken for bacterial cell count (log_10_ CFU/mL), and mixtures were incubated at 100 RPM and 28 °C for 24 h in an orbital incubator shaker (Innova 42, New Brunswick Scientific, USA). After the incubation time, a sample of the mixture was taken for the final bacterial count (log_10_ CFU/mL). Four criteria were considered to select the wall materials: cost, final bacterial cell count of *L. lactis* A12, dissolution (no presence of clumps), and ability to be added to high concentration (20% w/v or higher). The final cell count was compared to the control to determine if bacterial growth was statistically higher (positive), lower (negative), or did not change (equal).

Finally, the selected wall materials based on selection criteria were mixed with the culture medium containing probiotic bacteria to a final concentration of 40% w/v. The concentration of wall materials was determined according to the selected wall materials. Feed solution was prepared and spray-dried as described in section 2.6.1. Air pressure atomization was set at 1.5 bar. This mixture was selected for the experiment design.

It is important to highlight that even though *Priestia* species did not survive spray drying, it was decided to grow *L. lactis* A12 in co-culture conditions with *Priestia* species since this multistrain probiotic in a previous study exhibited higher antibacterial activity against *S. agalactiae* compared to *L. lactis* A12 alone [53]. Therefore, this work focused on improving the survival of *L. lactis* A12 during spray drying and exposure to gastrointestinal conditions.

### 2.6. Encapsulation of probiotic bacteria

#### 2.6.1. Preparation of feed solution.

The feed solution was prepared by mixing 200 mL of 17 h incubation culture medium containing probiotic bacteria (5.47% w/v solids content) with a mixture of the selected wall materials at different proportions (according to experiment design) at a final concentration of 40% w/v. The final solids content of the feed solution was 43.4% w/v. The final mixture was homogenized using a laboratory stirrer EURO-ST 20 HS (IKA®, USA) at 1600 RPM for 2 min. This final mixture was the feed solution. The initial bacterial count of *L. lactis* A12 was 8.65 ± 0.20 log_10_ CFU/mL, respectively.

#### 2.6.2. Spray drying of probiotic bacteria.

A pilot-scale spray-dryer (GEA Process Engineering Mobile Minor^TM^, GEA Niro, Dusseldorf, Germany) equipped with a pneumatic nozzle (1 mm diameter) was used to produce spray-dried probiotics. The feed solution was constantly homogenized using a magnetic stirrer (300 RPM), while was pumped with a peristaltic pump (MARLOW 520S, WATSON, Falmouth, UK) at an average flow rate of 40 mL/min. The atomizing air pressure was set according to the experiment design. Inlet and outlet temperatures of 180 and 90 °C, respectively were determined in previous experiments. The encapsulated probiotic was collected in a stainless-steel container, packed in aluminum bags under vacuum, and stored at 4 °C for further use.

#### 2.6.3. Characterization of spray-dried probiotics powders.

**Moisture content and water activity** Moisture content and water activity were determined according to the methodology described by Aragón-Rojas et al. [42].

**Viability of probiotic spray-dried powder** 1.0 g of spray-dried probiotic was added to 9 mL of Phosphate-buffer saline (PBS) solution. Then, serial dilutions were made. Viable cell count was performed by the plate count method in TSA at 28 °C after 24 h.

**Bacterial change cycle after drying** The spray-dried probiotic was reconstituted with distilled water until reached the initial solids content of 43.4% w/v. Then, the bacterial count was determined. The bacterial change cycle after drying (log_10_) was calculated as the difference between the bacterial count of reconstituted probiotics and feed solution [42].

**Bacterial change cycle under exposure to acid pH or bile salts***:* 1.0 g of spray-dried probiotic was mixed with 9 mL of simulated gastrointestinal solution (acid or bile salts) contained in a 50 mL falcon tube. Then, falcon tubes were agitated at 50 RPM and 28 °C for 2 h. After this, a sample of 1 mL from each falcon was taken and added to 9 mL of PBS. Then, serial dilutions were made. The final viable cell count was performed by the plate count method in TSA at 28 °C after 24 h. As a control, one gram of spray-dried probiotic was mixed with 9 mL of saline solution (0.89% w/v) [52].

The acid-simulated solution was prepared by adding HCl solution to BHI broth until pH 3 was reached. Bile solution was prepared by adjusting BHI broth to pH and adding a bile salt combination (Sigma Aldrich, St. Louis, MO, USA) to a concentration of 0.30% w/v. Both solutions were sterilized at 121 °C for 15 min [52].

The bacterial change after drying, exposure to acid pH, and bile salts was calculated using the following formula:

$Bacterialchange\left(\log_{10}\right)=Finalconcentrationofviablecell(\log_{10}\frac{CFU}{mL}-Initialconcentrationofviablecell\left(\log_{10}\frac{CFU}{mL}\right)$

#### 2.6.4. Experiment design.

The spray-drying design was carried out using an l-optimal combined design using the statistical software Design Expert v.10 (Stat-Ease Inc., Minneapolis, MN, USA). The design consisted of 19 runs with 3 duplicates and a triplicate in the central point mixture. The mixture components were the selected wall materials in section 2.5. Wall material A: 10–30% w/v and wall material B: 10–30% w/v. The process factor was the air pressure (1.0–1.5 bar). The response variables were moisture content (%), water activity (a_w_), bacterial change cycles after drying (log_10_), bacterial change cycles after exposure to pH 3 (log_10_), and bile salts (log_10_).

Model selection was carried out by considering the lowest *p*-value for the mixture and process factors, and the corrected Akaike information criterion (AIC_c_). ANOVA was carried out with a significant level of 0.05. Also, model reduction for the selected models was carried out to improve statistical parameters. ANOVA assumptions such as homoscedasticity, normality, and data independence were verified using residuals vs. predicted, normal probability (%) vs. residuals, and residuals vs. run number plots, respectively.

The best conditions of mixture and pressure in the spray drying process that maximize bacterial survival after drying and exposure to gastrointestinal conditions were achieved using the desirability function [57]. The criterion of desirability is a general approach in which the value of each response variable is transformed into a measurement ranging from 0 to 1; values close to 1 represent maximization processes, while values close to 0 represent minimization processes [52,58]. Validation of response variables was performed at optimal conditions. The error percentage of predicted and experimental data at the optimal conditions was calculated. Validation runs were performed in triplicates.

### 2.7. Comparison of SD and CM probiotic powders

Under optimal conditions from the encapsulation process (section 2.6.2.), the culture medium with grown bacteria with and without adding wall materials was spray-dried to obtain encapsulated (SD) and non-encapsulated (CM) probiotics, respectively.

#### 2.7.1. Probiotic properties of spray-dried probiotic.

Moisture content (%), water activity (a_w_), bacterial change cycles after drying (log_10_), bacterial change cycles after exposure to pH 3 (log_10_), and bile salts (log_10_) were evaluated for both powders [42].

#### 2.7.2. Antibacterial activity of spray-dried probiotic.

Individually, 1.0 g of each spray-dried probiotic (SD and CM) was added to 9 mL of PBS in a 15 mL-falcon tube, which was incubated at 28 °C for 17 h. Then, the final mixture was used to evaluate antibacterial activity against *Streptococcus agalactiae* according to the methodology described by Valle-Vargas et al. [52].

#### 2.7.3. Differential Scanning Calorimetry (DSC) and Thermogravimetric Analysis (TGA).

DSC and TGA analysis of spray-dried powders and wall materials was carried out following the methodology reported by Barón et al. [30] with some modifications.

Individually, 12–14 mg of SD, CM, whey powder, and maltodextrin were placed in sealed aluminum capsules and subjected to thermal analysis using a differential scanning calorimeter DSC 3 + Stare System (Mettler Toledo, Switzerland). The glass transition temperature (T_g_) and endothermal or exothermal transitions were studied using the STARe evaluation software. The thermal scan was performed in the following order: isotherm at −70 °C for 5 min; heating from −70 to 160 °C at 10 °C/min.

The thermal stability of SD, CM, whey powder, and maltodextrin was studied in a TGA/DSC Star 1 System (Mettler Toledo, Switzerland). Each powder (10 mg) was continuously weighed and heated from 20 to 450 °C at a heating rate of 10 °C/min.

DSC and TGA tests were carried out under a dynamic nitrogen inert atmosphere with a flow rate of 50 mL/min.

#### 2.7.4. Surface area and porosity by gas adsorption isotherms.

Porous structure of SD and CM powders were characterized using N_2_ and CO_2_ adsorption isotherms at 77 K and 273 K, respectively using a TriStar II PLUS gas sorptometer (Micromeritics^®^, USA) according to the methodology described by Barón et al. [30]. The specific area and pores width (micropores and mesopores) were estimated by the BET (Brunauer–Emmett–Teller) equation. Also, pore size distribution (PSD) was obtained. The PSD allows identified the cumulative and differential increase of specific surface and pore volume.

#### 2.7.5. FTIR.

Attenuated Total Reflectance–Fourier Transform-Infrared Spectroscopy (ATR-FTIR) was carried out according to the methodology described by Rojas-Muñoz et al. [59], increasing the number of scans (64). Spectra were processed by using the free-license Spectragryph v1.2.16.1 software (developed by Dr. Friedrich Menges, Oberstdorf, Germany). Spectra were baseline-corrected (adaptative correction; coarseness: 50; offset 0) and were normalized between 0 and 1 only for figure presentation. Deconvolution was performed by Omnic Spectra (Thermo Scientific).

#### 2.6.6. Hygroscopicity.

The hygroscopicity of SD and CM powders was determined using the methodology described by Barón et al. [30]. Individually, 1.0 g of each powder was placed in a pre-weighed aluminum capsule. Samples were placed in a desiccator with a saturated solution of NaCl (75% relative humidity) for 7 days. After that, the samples were weighed and the hygroscopicity was expressed as grams of adsorbed moisture per 100 g of sample (%).

#### 2.7.6. Statistical analysis.

Analysis of Variance (ANOVA) was conducted with a significance level of 0.05 for comparison of the viability of *L. lactis* A12 for wall materials selection. ANOVA assumptions such as homoscedasticity, normality, and data independence were verified using residuals vs. predicted, normal probability (%). Mean values were compared using a Least significant difference (LSD) test with alpha level of 0.05. Also, comparisons between two samples for probiotic properties and antibacterial activity were carried out using a two-tailed *t -test* with an alpha level of 0.05. Also, the homogeneity of variance for *t-test* was confirmed with a *F- test* (alpha level of 0.05).

### 2.8. Stability of spray-dried probiotic and spray-dried probiotic supplemented in fish feed during storage conditions

Encapsulated probiotic (SD) was used for the stability study under storage conditions [42]. The spray-dried powder was packed in aluminum bags, vacuum-sealed (0.0009 MPa, C200; Multivac, Barcelona, Spain), and stored at 4, 25 and 37 °C for 60 days. Viable cell count, water activity, and water content were determined every 5 days. As a control, CM powder was stored at the temperature with the highest survival rate for SD during 60 days of storage.

Additionally, a second storage experiment was conducted by simulating the conditions of opened powders. Then, CM and SD powders were packed without vacuum and stored at the temperature with the highest survival rate for 60 days. For this, the same package was open and closed to take a sample at different time during storage. As control, both powders were packed under vacuum conditions and open to sample after 60 days of storage to determine survival rates.

Commercial fish feed MOJARRA 45 in powder form (Italcol, Colombia) was mixed with SD probiotic powder to obtain an initial viable cell count of 5.53 ± 0.03 log_10_ CFU/g of feed of *L. lactis A12* and moisture content of 5.64 ± 0.25%. The final mixture was vacuum-packed and stored as described in this section. Viability was determined for 60 days.

Data of viability from the storage study was plotted as bacterial viability loss vs time according to the methodology described by Aragón-Rojas et al. [42] in order to calculate the bacterial viability loss specific rate (*k*_*m*_) at the evaluated temperatures.

## 3. Results and discussion

### 3.1. Selection of wall materials

Table 1 shows the results of final cell count of *L. lactis* A12 and *Priestia* species grown with different wall materials. As the first criterion, the final cell count was compared to the control to determine whether the bacterial growth was positive, negative, or did not change. *L. lactis* A12 grew with maltodextrin and sodium alginate. The other materials did not affect negatively bacterial growth. *Priestia* species were affected negatively by maltodextrin, gum arabic, and whey, but it is bacterial growth increased when sodium alginate modified starch and gelatin were used.

**Table 1:** Results of wall materials selection.

Wall material (% w/v)	*Priestia* species (log_10_ CFU/mL)	*L. lactis* A12 (log_10_ CFU/mL)	Final pH	Cost ($USD/kg)	Dissolution (presence of clumps)
Control	6.76 ± 0.04^b^	9.35 ± 0.18 cd	6.5–7.0	–	No
High methoxy pectin (2%)	6.69 ± 0.07^b^	9.31 ± 0.02^bc^	6.0–6.5	21.64	Yes
Whey (40%)	6.46 ± 0.10^c^	9.24 ± 0.12^bc^	5.5–6.0	3.08	No
Gum arabic (40%)	0.00 ± 0.00^d^	8.93 ± 0.24^d^	4.5–5.0	14.64	Yes
Maltodextrin (40%)	0.00 ± 0.00^d^	9.59 ± 0.05^a^	4.5–5.0	2.20	No
Sodium caseinate (1%)	6.66 ± 0.01^b^	9.30 ± 0.04^bc^	7.0–7.5	15.98	No
Sodium alginate (4%)	6.89 ± 0.01^a^	9.39 ± 0.11^b^	7.0–7.5	32.67	Yes
Starch (20%)	6.71 ± 0.03^b^	9.20 ± 0.06 cd	6.5–7.0	2.77	Yes
Modified starch (20%)	6.94 ± 0.05^a^	9.28 ± 0.07^bc^	6.5–7.0	3.50	No
Gelatin (4%)	6.92 ± 0.08^a^	9.18 ± 0.02 cd	7.0–7.5	15.45	No

Difference superscript lower-case letters within column indicate significant differences (*p < 0.001*) using LSD test.

Positive bacterial growth of *L. lactis* A12 in maltodextrin could be attributed to genes related to maltose and maltodextrin metabolism [49], particularly the maltodextrin glucosidase gene which liberates glucose from the reducing end of maltodextrin [60]. *Bacillus* species, specially *B. subtilis* have been reported for their ability to utilize maltose and maltodextrin derived from polysaccharides, like starch or glycogen [61]. Positive bacterial growth of *L. lactis* A12 and *Priestia* species using alginate as nutrient source could be associated that some microorganisms including bacteria could use marine-derived polysaccharide as carbon source by degrading alginate using alginate lyases [62,63], which results in oligosaccharides and monosaccharides that can be metabolized by bacteria [64–66].

Negative bacterial growth exhibited with gum arabic for *Priestia* species could be related to the fact that gum arabic in aqueous solutions presents low pH (4.21–4.96) [67], which could inhibit bacterial growth of *Priestia* species. This agrees with *Priestia* species growing in co-culture with *L. lactis* A12, where *Priestia* species did not survive with a final pH of 4.5–5.0*. L. lactis* exhibits higher nutrient consumption in pH values around 5.8–6.5 [68], while values lower than 4.0 inhibit bacterial growth [69]. On the other hand, *Bacillus* species grow within pH between 5.5 and 8.0 [70–72]. *L. lactis* has the ability to metabolize sugars and convert them into lactic acid [73], which reduces medium pH resulting in the inhibition of nutrient consumption and bacterial growth [74].

The second criterion to consider was dissolution. Dissolution in the culture medium with growing bacteria was evaluated since this is the medium where wall materials will be dissolved. Gum arabic, pectin, starch, and sodium alginate were difficult to dissolve and presented clumps that could obstruct the spray dryer atomizer device; therefore, they were not considered.

Taking into account the four criteria, including cost (2.20–3.50 $ USD/kg) and ability to form a solution with 20%w/v solids or higher, whey, maltodextrin, and modified starch were selected. High solubility and low viscosity at high solids concentration in aqueous solution are desired parameters to facilitate feed preparation and atomization in spray drying [75]. Additionally, a high concentration of solids in feed solution is desired by industry because of the improvement of drying yields, lower energy cost, and higher survival of probiotic bacteria [24,76].

Whey (WH) was mixed in binary mixtures with maltodextrin (MD) and modified starch (MS) in proportion WH:MD and WH:MS of 0.46: 0.54 w/w to a final concentration of 40% w/v of solids content in the feed solution. The probiotic powder from the WH:MD mixture presented a higher viable cell count of *L. lactis* A12 (6.88 ± 0.13 log_10_ CFU/g) than the WH:MS mixture (6.17 ± 0.12 log_10_ CFU/g). Therefore, the mixture of whey/maltodextrin was selected for the experiment design and bacterial reduction after drying, exposure to pH 3, and bile salts only were considered *L. lactis* A12 cell counts.

### 3.2. Encapsulation of probiotic bacteria by spray-drying

#### 3.2.1. Model fitting of l-optimal design.

Response variables data of the l-optimal combined design for spray-dried probiotic characteristics are shown in Table 2. Analysis of Variance is shown in Table 3.

**Table 2:** BBD with experimental results of spray-drying process.

Run	A: Whey (% w/v)	B: Maltodextrin (% w/v)	C: Pressure (bar)	Moisture content (%)	Water activity (a_w_)	Bacterial change after drying (log_10_)	Bacterial change in pH 3 (log_10_)	Bacterial change in bile salt (log_10_)
1	25	15	1.1	1.18 ± 0.09	0.1999 ± 0.0066	-1.97 ± 0.01	-0.24 ± 0.02	-0.10 ± 0.02
2	10	30	1.3	1.13 ± 0.16	0.1822 ± 0.0022	-2.45 ± 0.07	-2.03 ± 0.04	-1.28 ± 0.05
3	30	10	1.0	2.04 ± 0.38	0.2338 ± 0.0095	-1.66 ± 0.04	-0.69 ± 0.04	-0.75 ± 0.02
4	20	20	1.5	2.32 ± 0.16	0.1772 ± 0.0043	-2.49 ± 0.04	-0.41 ± 0.02	0.26 ± 0.07
5	10	30	1.3	2.27 ± 0.35	0.1993 ± 0.0015	-2.43 ± 0.07	-2.05 ± 0.06	-1.35 ± 0.06
6	30	10	1.5	1.97 ± 0.10	0.2037 ± 0.0056	-2.26 ± 0.04	-0.06 ± 0.00	0.29 ± 0.00
7	20	20	1.3	2.11 ± 0.53	0.1925 ± 0.0059	-2.15 ± 0.01	-0.37 ± 0.09	0.53 ± 0.05
8	15	25	1.0	1.89 ± 0.57	0.1962 ± 0.0037	-2.14 ± 0.04	-1.32 ± 0.23	-0.63 ± 0.18
9	20	20	1.5	1.49 ± 0.17	0.1742 ± 0.0074	-2.51 ± 0.08	-0.42 ± 0.02	0.24 ± 0.03
10	15	25	1.1	2.12 ± 0.15	0.1987 ± 0.0019	-2.56 ± 0.07	-0.69 ± 0.05	-0.27 ± 0.09
11	30	10	1.3	2.14 ± 0.15	0.2221 ± 0.0037	-1.81 ± 0.08	-0.09 ± 0.04	-0.02 ± 0.00
12	30	10	1.3	2.22 ± 0.21	0.2223 ± 0.0070	-1.68 ± 0.05	-0.08 ± 0.02	-0.02 ± 0.00
13	20	20	1.3	2.33 ± 0.36	0.1897 ± 0.0030	-2.41 ± 0.16	-0.25 ± 0.03	0.33 ± 0.08
14	20	20	1.0	2.52 ± 0.06	0.1993 ± 0.0050	-2.22 ± 0.04	-0.11 ± 0.02	0.05 ± 0.01
15	20	20	1.3	2.87 ± 0.43	0.2267 ± 0.0032	-1.68 ± 0.03	-0.34 ± 0.02	-0.20 ± 0.03
16	25	15	1.4	1.99 ± 0.21	0.1659 ± 0.0020	-1.19 ± 0.01	-1.21 ± 0.09	-0.01 ± 0.00
17	10	30	1.5	2.26 ± 0.23	0.1908 ± 0.0021	-2.54 ± 0.01	-0.90 ± 0.04	0.24 ± 0.10
18	15	25	1.4	2.16 ± 0.06	0.1772 ± 0.0025	-2.13 ± 0.12	-0.98 ± 0.09	-0.38 ± 0.05
19	10	30	1.0	2.69 ± 0.26	0.2100 ± 0.0039	-2.66 ± 0.02	-2.00 ± 0.20	-0.46 ± 0.14

**Table 3:** ANOVA and statistical parameters of spray-drying process.

	Bacterial change after drying	Bacterial change after pH 3	Bacterial change after bile salts
Model	0.0052	0.0003	0.0022
Linear mixture	0.0005	< 0.0001	0.0053
C	–	0.3328	–
AB	–	0.0163	0.0017
AC	0.0737	–	0.0229
BC	0.6317	–	0.0533
C^2^	–	–	–
ABC	–	–	0.2063
AC^2^	0.1336	–	0.5821
BC^2^	–	–	0.0014
ABC^2^	–	–	0.0515
AC^3^	0.0380	–	–
BC^3^	0.6288	–	–
Lack-of-Fit	0.5375	< 0.0001	0.3537
R^2^	0.7873	0.7084	0.8520
R^2^_adjusted_	0.6520	0.6501	0.7444
Adequate Precision	7.2031	9.0385	8.7840

A: Whey, B: Maltodextrin; C: Pressure

The moisture content of spray-dried bacteria was not affected (*p = 0.9260*) by mixture and process parameters. On the other hand, water activity (*p = 0.0129)*, bacterial change after drying (*p = 0.0052*), pH 3 (*p = 0.003*), and bile salts (*p = 0.0022*) were influenced by mixture and/or process parameters of the spray-drying process. Model for bacterial reduction after drying and bacterial change after bile salts presented R^2^ values higher than 0.70, which indicates that can be used to predict response variables.

Equations (1) and (2) show the fitting of the significant models expressed in terms of actual and factor levels.

**Table:** 

$Bacterialchangeafterdrying\left(\log_{10}\right)=6.90\times [A]-1.06\times [B]-17.24\times [AC]+2.39\times [BC]-14.12\times \left[AC^{2}\right]-1.91\times \left[BC^{2}\right]-3.81\times \left[AC^{3}\right]+0.50\times \left[BC^{3}\right]$	(1)
$Bacterialchangeafterbilesalts\left(\log_{10}\right)=0.45\times [A]+2.01\times [B]-0.13\times [AB]-0.93\times [AC]-3.50\times [BC]+0.23\times [ABC]+0.42\times \left[AC^{2}\right]+1.44\times \left[BC^{2}\right]-0.09\times \left[ABC^{2}\right]$	(2)

A: Whey (%w/v), B: Maltodextrin (%w/v), and C: Pressure (bar).

#### 3.2.3. Influence of mixture components and process parameters on probiotic characteristics.

**Fig 1:**
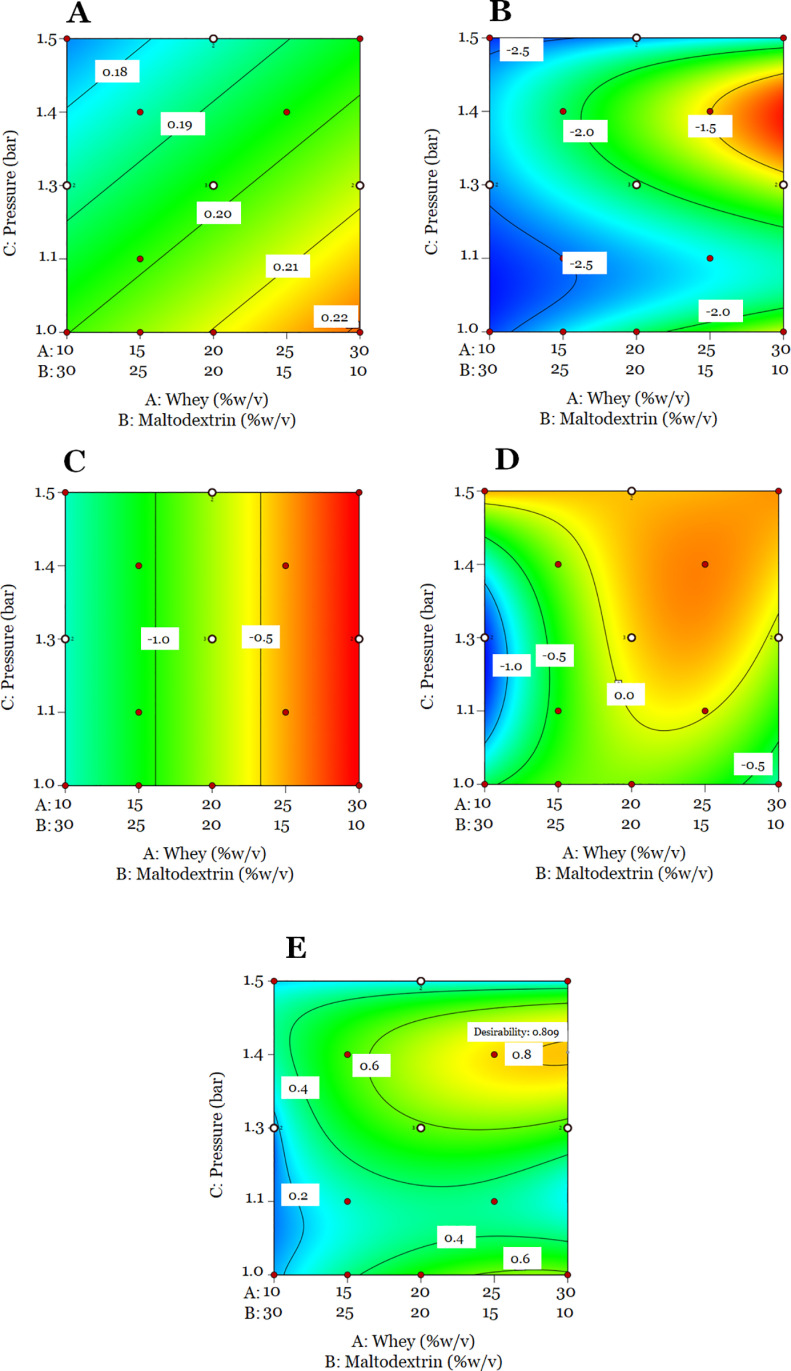
Contour plots of response variables of spray-drying process. (A) water activity, (B) bacterial reduction after drying, (C) bacterial change after pH 3.00, (D) bacterial change after bile salts, and (E) desirability function.

Fig 1 shows the contour plots of response variables and the desirability function. Moisture content values varied from 1.10 to 2.85%. However, the water activity of the probiotic powder (0.1659–0.2267 a_w_) was influenced by atomization pressure as shown in Fig 1A. It can be observed that higher atomization pressure led to lower water activity. This could be associated that higher atomization pressure produced smaller particles that have higher evaporation rates leading to lower water activity values [77]. Food powders with moisture content and water activity lower than 4% and 0.6, respectively, are more stable during storage due to inhibiting the growth of undesirable microorganisms and chemical reactions [35,42].

Bacterial reduction after drying was influenced by the maltodextrin/whey ratio and atomization pressure. Lower bacterial reductions after drying were achieved when a higher concentration of whey was used in the wall material mixtures (Fig 1B). Also, it can be observed that higher pressure leads to higher protection for the probiotic bacteria after drying, exhibiting its lowest reduction (lower than -1.51 log_10_) at approximately 1.4 bar. Bacterial reduction after drying ranged from -2.66 to -1.19 log_10_, which corresponds to survival rates of spray-dried probiotics between 69.25 and 86.24%. Survival of probiotic bacteria after exposure to pH 3 solution ranged from 65.58 to 98.93%, which corresponds to bacterial reduction between -2.05 and -0.06 log_10_, respectively (Fig 1C). A higher survival rate was observed at a higher proportion of whey in the mixture. Finally, survival rates after bile salts exposure were higher (89.09–100%) than in acidic conditions. In bile salts, probiotic bacteria exhibited higher reductions (close to -1.35 log_10_) at a high concentration of maltodextrin and atomization pressure between 1.0 and 1.4 bar (Fig 1D), while lower reductions were observed at a high proportion of whey (> 20% w/v) and atomization between 1.1. and 1.5 bar. Bacterial reductions after drying reported in this study are similar to those reported for the spray-drying of *L. fermentum* K73 (-1.90 to 6.50 log_10_) using maltodextrin and whey as wall materials [42].

In general, the high proportion of whey (20–30% w/v) and high atomization pressure (1.3–1.5 bar) improved probiotic survival after drying, exposure to pH 3, and bile salts. A possible explanation for this is that during drying, whey proteins suffer thermal denaturation [78] that cause the unfolding of whey proteins (being beta-LG the major protein) and exposure of reactive amino acids causing the irreversible aggregation of proteins that could have a protective effect in probiotic bacteria against adverse factors like drying, acid, and bile salts environments [79]. Also, the presence of alpha-LA increased the proportion of very large aggregates [80]. Another possible explanation for the protective effect of whey proteins under exposure to bile salts is the ability of whey proteins to binding bile salts through hydrophobic interactions [81], preventing bile salts to interact with probiotic bacteria contained in the spray-dried microcapsules, avoiding the damage cell membrane which results in cell death [82].

#### 3.2.4. Optimization and model validation.

Bacterial change after drying and exposure to bile salts were the response variables selected to be optimized, since they showed the highest R^2^ values and fitted the experimental data. The optimal conditions that maximize the selected variables were: 30% w/v whey, 10% w/v maltodextrin, and 1.4 bar with a desirability value of 0.879 (Fig 1E). Desirability values higher than 0.7 indicate a good optimization of experimental data of each response variable [30]. Predicted values for bacterial change after drying and exposure to bile salts were -1.22 and 0.14 log_10_, respectively. The experimental error for bacterial reduction after drying was 9.83%. Experimental error values lower than 10% indicate that the desirability function was a useful statistical tool for the optimization of spray drying parameters.

### 3.3. Comparison of encapsulated and non-encapsulated *L. lactis* A12

Results of probiotics characteristic of encapsulated and non-encapsulated bacteria are shown in Table 4. *L. lactis* A12 in SD powder exhibited higher survival after drying *(p < 0.001*) and exposure to bile salts (*p < 0.001*) than CM powder. It is important to highlight that *L. lactis* A12 in CM powders did not survive after exposure to bile salts. However, *L. lactis* A12 had a higher survival *(p = 0.*0117) in CM powder under exposure to pH 3. Water activity (*p = 0.2512*) and moisture content (*p = 0.2972*) between SD and CM powder presented similar values.

**Table 4:** Probiotic characteristics of CM and SD powders.

	CM	SD
Bacterial change of *L. lactis* A12 after drying (log_10_)	-3.31 ± 0.06^b^	-1.34 ± 0.12^a^
Bacterial change of *L. lactis* A12 in pH 3 (log_10_)	0.12 ± 0.03^a^	-0.34 ± 0.06^b^
Bacterial change of *L. lactis* A12 in bile salts (log_10_)	-6.42 ± 0.10^b^	-0.25 ± 0.00^a^
Moisture content (%)	1.57 ± 0.24^a^	1.34 ± 0.22^a^
Water activity (a_w_)	0.1512 ± 0.0003^a^	0.1466 ± 0.0041^a^

SD: encapsulated and CM: non-encapsulated probiotic bacteria. Different superscripted letters (a-b) between rows indicate significant difference (*p *< *0.05*) using a two-tailed *t t*est.

The use of wall materials improves bacteria survival during drying due to whey and maltodextrin creating a compact particle [42,83] that protects probiotic bacteria from external factors such as high temperatures, oxygen exposure, and bile salt presence [24,84]. In particular, bile salts can interact with cell membranes due to their detergent-like properties, dissolving them, causing DNA damage, and finally leading to cell death [82].

#### 3.3.1. Antagonistic activity.

SD powder probiotics exhibited higher (*p < 0.001*) antagonism than CM against *S. agalactiae* (Table 5). After 17 h of incubation, *L. lactis* A12 in CM powder was not observed. The opposite situation occurred in SD powder, when *L. lactis* A12 was viable with a final cell count of 9.11 ± 0.45 log_10_ CFU/mL. A possible explanation could be that *L. lactis* A12 in CM powders had fewer nutrients available for consumption since in the final culture medium there was no presence of sucrose and glucose, however, lactose, xylose, galactose, fructose, lactic acid, and acetic acid were present in a concentration of 1.46, 1.54, 0.09, 1.52, 1.72, and 0.59 g/L, respectively. On the other hand, SD powders have maltodextrin (glucose) as the main carbon source, leading to bacterial growth after incubation. Related to antagonistic activity of encapsulated *L. lactis* A12 against fish pathogen *S. agalactiae* could be associated with cell viability after the incubation period, hence the bacteria could exhibited antagonism against targeted microorganisms through the production of bacteriocin and/or bacteriocin-like peptides with antibacterial activity [85]. *L. lactis* A12 exhibited antibacterial activity against this pathogen in previous studies at *in vitro* and *in vivo* level [50,53,86]. This bacteria have a gene related to the production of the Lactococcin family bacteriocin [49].

**Table 5:** Antibacterial activity of spray-dried probiotic.

	Initial cell count	Final cell count	AA
	(log_10_ CFU/mL)	(log_10_ CFU/mL)	(mm)
CM	6.38 ± 0.10^a^	0.00 ± 0.00^b^	0.00 ± 0.00^b^
SD	6.38 ± 0.05^a^	9.11 ± 0.45^a^	9.83 ± 0.52^a^

SD: encapsulated and CM: non-encapsulated probiotic bacteria. Different superscripted letters (a-b) between rows indicate significant difference (*p *< *0.05*) using a two-tailed *t t*est.

#### 3.3.2. Thermal stability of probiotic powders and wall materials.

**Fig. 2:**
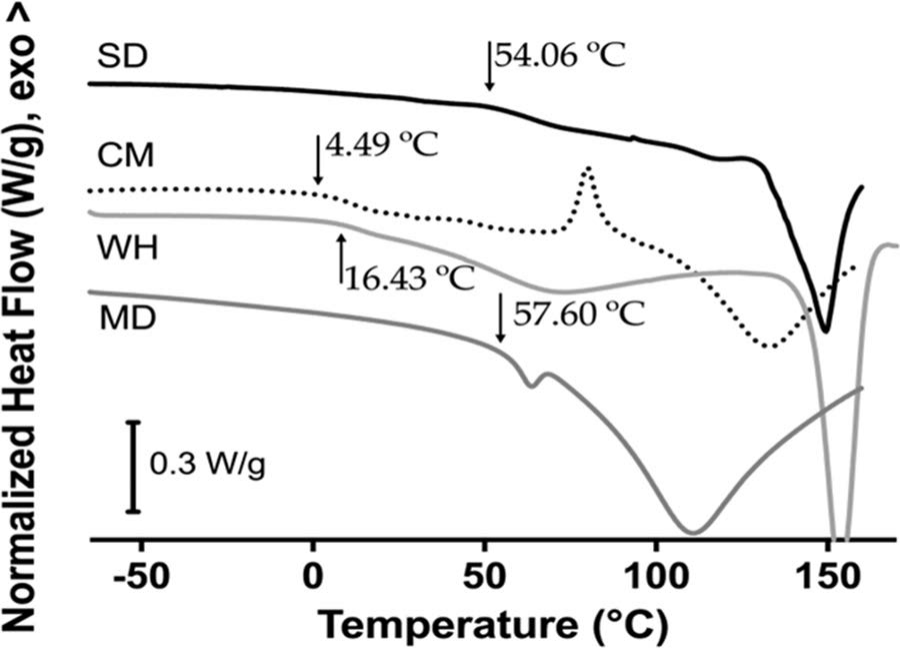
DSC curves of spray-dried powders and wall materials. SD: encapsulated probiotic, CM: non-encapsulated probiotic, WH: whey, and MD: maltodextrin.

Fig 2 shows the DSC curve of whey, maltodextrin, CM, and SD powders. In DSC curves, the black arrow represents the onset temperature of the glass transition of each sample, which indicates the beginning of the transition from glassy to rubbery state. The midpoint values of the glass transition (T_g_) for whey, maltodextrin, CM, and SD powders were 22.81, 61.20, 12.65, and 64.44 °C respectively. Endothermic peaks after T_g_ for WH (152.42 °C), and SD (149.45 °C) [87,88], MD (110.81 °C), CM (132.77 °C), could be related to the evaporation of water and volatile compounds [30,89]. For CM powder, this peak could also be the melting point (109–154 °C) of sugars such as xylose, galactose, and fructose that are present in this sample. In the whey sample, a small endothermic peak between 60 and 80 °C could be related to the denaturation of whey proteins [78]. The exothermic peak (79.85 °C) in CM sample could be related to the crystallization of xylose, fructose, and/or galactose present in this powder [90].

Besides the obvious implications regarding the higher T_g_ value of SD concerning CM, which allows to storage of the powder above ambient temperature minimizing physicochemical changes due to the glassy state.

**Fig 3:**
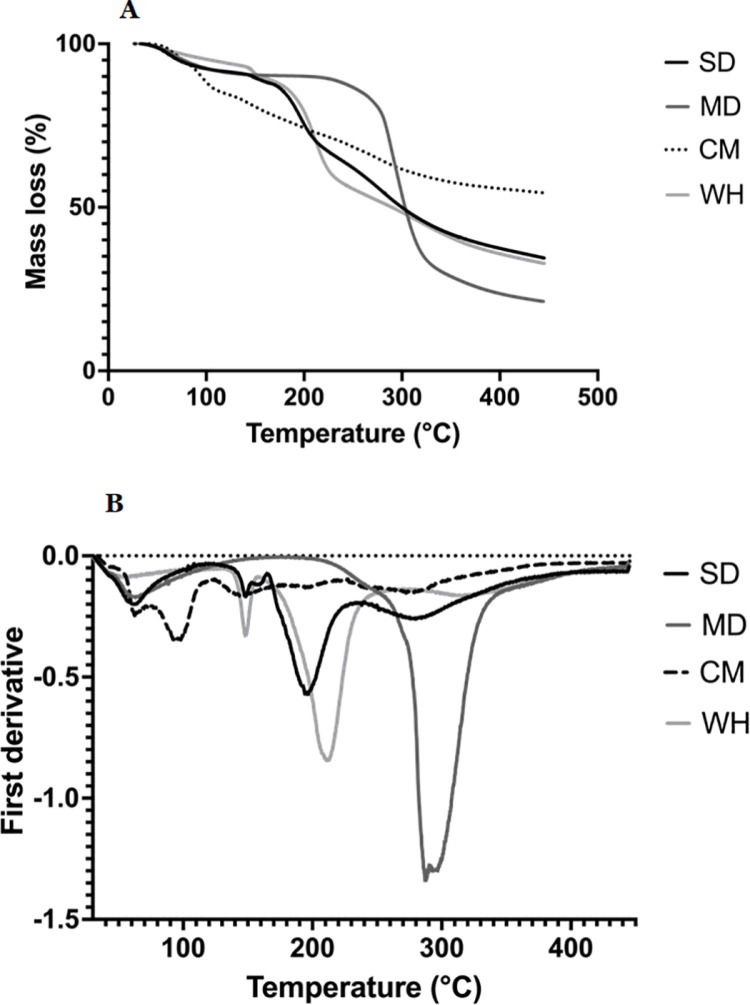
(A) TGA and (B) DTGA curves of spray-dried powders and wall materials. SD: encapsulated probiotic, CM: non-encapsulated probiotic, WH: whey, and MD: maltodextrin.

Fig 3A shows the TGA curves of the samples during heating. The thermal decomposition of samples is related to their chemical composition, in Fig 3A it can be observed that the lowest and highest mass loss is presented in CM (45.63%) and MD (76.85%), respectively. SD (65.51%) sample exhibits a similar profile of mass loss to WH (67.02%), this is expected since SD sample is composed approximately of 73.81% WH. The major loss for all samples is presented in the second region with onset temperature of thermal decomposition for CM, SD, MD, and WH of 120.14, 164.66, 234.37, and 167.43 °C, respectively. The last region for all samples could be associated to the decomposition and polymerization of carbon structures [91].

From the first derivate (DTGA) curve (Fig 3B), it can be observed that for CM and MD samples, peaks lower than 100 °C could be related to volatile and water evaporation [89]. While SD (59.7 °C) and WH (48.2 °C) could be related to volatile compounds. Peaks at 148.77, and 156.30 °C could be associated to water evaporation binding with lactose in SD and WH samples, respectively [87,88,91,92]. The second region for all samples is the related decomposition of di- and polysaccharides, proteins, and amino acids present in the samples [93,94]. The thermal decomposition temperatures for CM, SD, WH, and MD were 144.35, 196.21, 211.68, and 287.66 °C, respectively [87,95]. The final region for all samples is attributed to the decomposition of organic compounds present in the samples [30]. The displacement and size of the peaks are related to the composition of the samples, especially in SD powder, which is composed of 73.81% WH, 27.77% MD, and 1.58% water.

#### 3.3.3. Analysis of porous structure.

**Fig 4:**
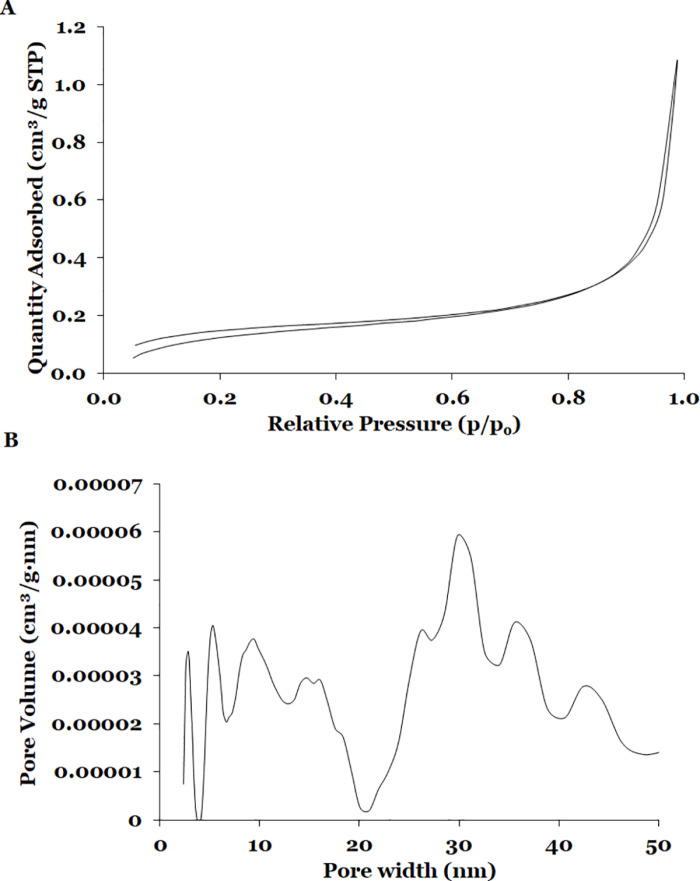
Analysis of porous structure of SD sample. **(a)** N_2_ adsorption isotherm and **(b)** Pore Size Distribution.

The porous structure of CM and SD powders was evaluated by sorption isotherms with two gasses, BET area, and pore size distribution. No interaction between CO_2_ and powder sample indicates that both materials are not microporous. Similarly, N_2_ did not interact with CM sample. On the other hand, SD sample absorbed N_2_ and exhibited a type II isotherm (Fig 4A), which is a characteristic of materials with non-significant micro or meso-porosity or for macroporous adsorbents. The shape is the result of unrestricted monolayer-multilayer adsorption up to high relative pressure [96]. It has been reported that milk powders are non-porous materials (regarding micro and meso-porous) and exhibit the same type of isotherm [97]. Fig 4B shows the pore size analysis of SD sample which exhibited a bimodal distribution (2–20 nm and 20–50 nm) with low pore volumes (0.00001–0.00006 cm^3^/g ∙ nm). Finally, the BET superficial area of SD was low (0.5219 m^2^/g). Milk powders (0.1–0.6 m^2^/g) have low superficial area [97]. Similar values (0.27–2.28 m^2^/g) have been reported for food matrices [30,98–101].

#### 3.3.4. ATR - FTIR.

**Fig 5:**
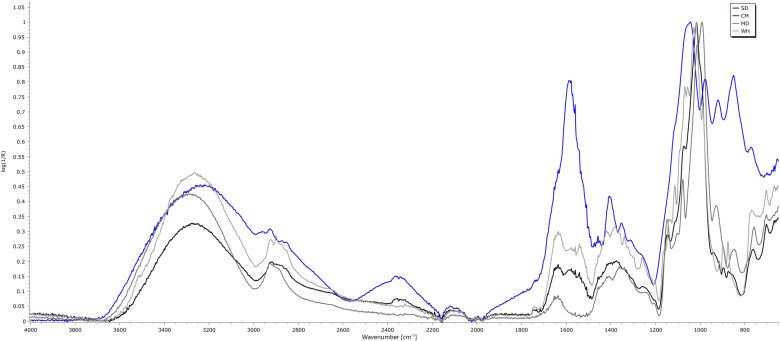
FTIR spectra of spray-dried powders and wall materials. SD: encapsulated probiotic, CM: non-encapsulated probiotic, WH: whey, and MD: maltodextrin.

Fig 5 shows the FTIR spectra. The first thing noticed was the resemblance of SD spectrum to WH and MD, sharing several common bands, even though with some displacement, as will be further analyzed. WH shows their main absorption bands at 1029, 1018, and 988 cm^-1^; MD, at 1077, 1014, and 992 cm^-1^. The main band of SD at 1018 cm^-1^ seems a combination of WH and MD; however, at least three main bands were obtained by deconvolution (at 1076, 1023, and 989 cm^-1^), which were attributed to MD, a combinatorial band and MD and WH, respectively. This region is typically characterized by the presence of C-O-C groups, glycosidic linkages, and alpha and beta configurations of monosaccharides [102]. On the other hand, CM showed a very distinctive pattern, indicative of its saccharide composition (mainly xylose, fructose, and lactose). Xylose showed strong absorption bands at 1040 and 1020 cm^-1^; fructose at 1095, 1080, 1050 (main peak), 1025, 975, and two shoulders at 1060 and 1030 cm^-1^; and lactose, at 1115, 1095, 1085, 1070, 1055, 1035 and 1015 (main peaks), 1005 and 987 cm^-1^ (SpectraBase, John Wiley & Sons, Inc.). CM showed 7 bands with peaks between 1118 and 771 cm^-1^. Then, the observed spectrum is a combination of the afore-mentioned bands, with some shifting, which could be produced by the interaction among components. Moreover, after deconvolution of CM spectrum, 11 peaks were identified, with maxima at 1181, 1125, 1074, 1036, 977, 922, 884, 852, 812, 766, and 716 cm^-1^; some of them corresponded directly to the peak (977, 922, 852 cm^-1^).

The OH stretching vibration in the region 3000–3600 cm^-1^ showed differences in the maximum peak position: MD (3284), SD (3273), WH (3264), CM (3239 cm^-1^), respectively. These positions followed the T_g_ values obtained by DSC (Fig 2). As stated by Wolkers et al. [103] the OH position has also been related to the average length of the hydrogen bonds, and hence to the molecular packing of the amorphous sugars. As T_g_ increases, sugars exhibit a decreased degree of molecular packing, which allows to rearrangement of hydrogen bonds during a temperature change in the glassy state enhancing the probable protection of the encapsulated agent. Finally, CM also shows a strong absorption peak at 1586 and another at 1405 cm^-1^ which could be related to the bending vibration of N-H groups [104].

#### 3.3.5. Hygroscopicity.

SD and CM samples absorbed 12.95 and 69.73% of their initial weight in water after 7 days of storage, showing a huge difference among them. HG values higher than 25% are considered extremely hygroscopic, while HG values lower than 10% are non-hygroscopic [105]. The high hygroscopicity of CM powder could be related to the presence of low molecular weight sugars increasing their ability to absorb water molecules [106]. On the other hand, CM sample presents N-H groups that could form hydrogen bonding with water molecules [107,108] increasing their ability to absorb water from the environment and hence increase hygroscopicity.

Overall, the adequate selection of wall materials provided the desirable characteristics for the designed powder: low hygroscopic powder with T_g_ above ambient temperature.

### 3.4. Stability of spray-dried probiotic and supplemented feed during storage

**Fig 6:**
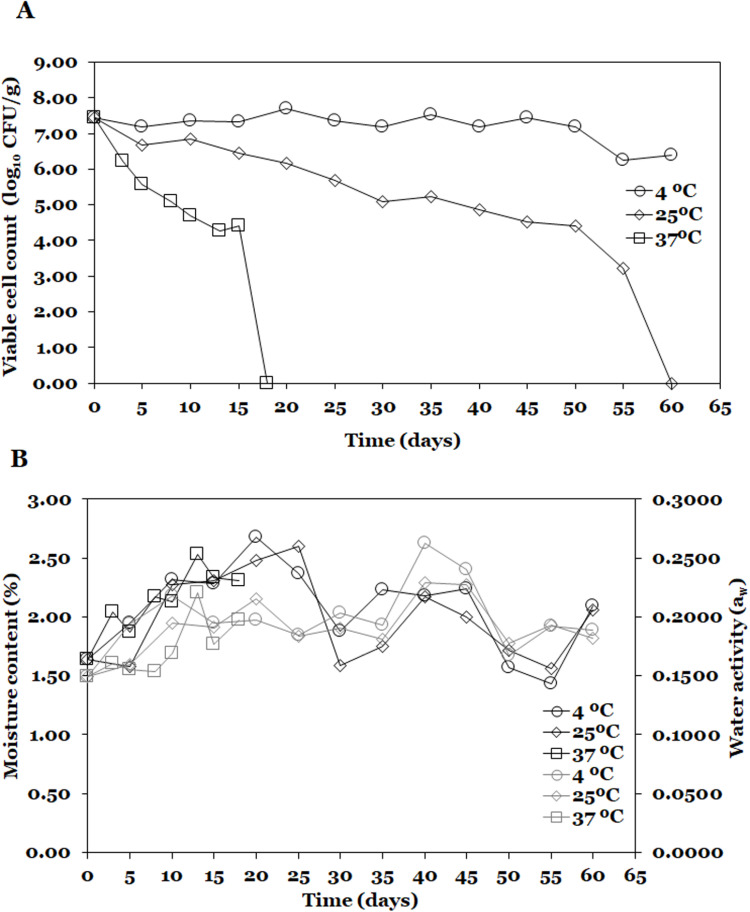
Stability of SD probiotic powder during storage at three temperatures. **(A)** viability and **(B)** moisture content and water activity. Dark and gray lines indicate moisture content and water activity, respectively.

Considering probiotic characteristics such as tolerance to bile salts and antagonistic activity of *L. lactis* A12, as well as physical properties such as glass transition temperature and hygroscopicity, encapsulated probiotic was used for the stability test at three temperatures (4, 25, and 37 °C). Viability, moisture content, and water activity results during storage are shown in Fig 6. On day 0, the viable count of *L. lactis* A12 was 7.44 ± 0.19 log_10_ CFU/g. After 20 days of storage, *L. lactis* A12 lost 100% of its viability at 37°C, while exhibiting a survival rate of 100 and 82.93%, respectively, at 4 and 25°C. After 60 days, *L. lactis* A12 did not survive at 25 °C, however, at 4 °C probiotic bacteria showed a final viable count of 6.39 ± 0.08 log_10_ CFU/g resulting in a survival rate of 85.88%. Probiotic bacteria exhibited higher cellular viability loss rate (*k*_*m*_, day^-1^) at higher storage temperatures with values of -0.0163, -0.0804, and -0.3053 day^-1^ for 4, 25, and 37 °C, respectively.

The initial moisture content and water activity were 1.64 ± 0.14% and 0.1489 ± 0.0059 a_w_, respectively. After 60 days moisture content for probiotics storage at 4 and 25 °C was 2.09 ± 0.14 and 2.06 ± 0.15%, respectively. Finally, water activity after 60 days for 4 and 25 °C was 0.1890 ± 0.0022 and 0.1819 ± 0.0105 a_w_, respectively.

**Fig 7:**
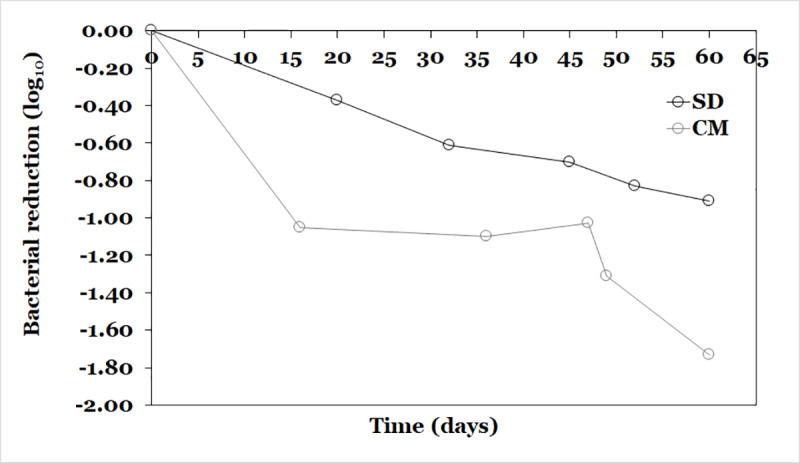
Evolution of viability of CM and SD probiotic powder during storage at 4 °C under non-vacuum conditions.

For the study of non-vacuum packing, initial cells count of *L. lactis* A12 in CM and SD powders was 7.55 ± 0.01 and 7.76 ± 0.19 log_10_ CFU/g, respectively. For CM powder, after 16 days its viability reduced to 6.50 ± 0.04 log_10_ CFU/g. Between day 16 and 47 *L. lactis* A12 maintained its viability stable, however, after day 47 and until day 60 viability decreased to 5.82 ± 0.07 log_10_ CFU/g, which results in a survival rate of 77.08% (Fig 7). In the case of SD powder, cell viability decreases linearly during the 60-day period of storage with a final cell count of 6.85 ± 0.06 log_10_ CFU/g, resulting in a survival rate of 88.27%. For encapsulated and non-encapsulated probiotic bacteria at 4 °C the *k*_*m*_ values were -0.0279 day^-1^ and 0.016 day^-1^, respectively. With respect to the packed powders under vacuum conditions, after 60 days of storage, CM and SD powder exhibited a survival rate of 100 and 93%, respectively. Even though CM probiotic powder vacuum packaged exhibited a higher survival rate than SD powder, when packaged probiotic is open and close constantly, what it is expected in the reality, CM powder loss its viability faster than SD powder according to results of *k*_*m*_ values for both powders. This could be related to the fact that during storage, exposure to oxygen could cause saturation and consequent oxidation of membrane of lipids that may exert a negative effect on viability [24]. Also, opening the package and the exposure to humidity during storage conditions could cause the absorption of water, being higher in CM powder which presented a higher hygroscopicity than SD powder, resulting in a possible decrease of T_g_ and viability of *L. lactis* A12 for both powders. It is reported that an increase of moisture content in food powders leads to lower T_g_ [91]. When storage temperature (T) is close T_g_ (T – T_g_ = 0) inactivation of dried cells could occurs, therefore it suggested to storage 50 °C below T_g_ to reduce completely molecular movement in amorphous solids [109]. This may reaffirm the importance of wall materials to protect probiotic bacteria against environmental factors during storage [110]. Also, it is important to highlight that both vacuum and non-vacuum-packaged SD probiotic exhibited similar behavior during storage, which is related to the low hygroscopicity of the designed powder. These results are in agreement with those reported by Jannah et al. [111] who supplemented instant coffee with encapsulated probiotic *L. plantarum* and stored with and without vacuum packaging at 4, 30, and 37 °C for 50 days. They found that probiotic-supplemented instant coffee vacuum packaged, and non-vacuum packaged at 4 °C presented similar loss viability constant (*k*_*m*_) with values of 0.0082 and 0.0091 day^-1^, respectively.

**Fig 8:**
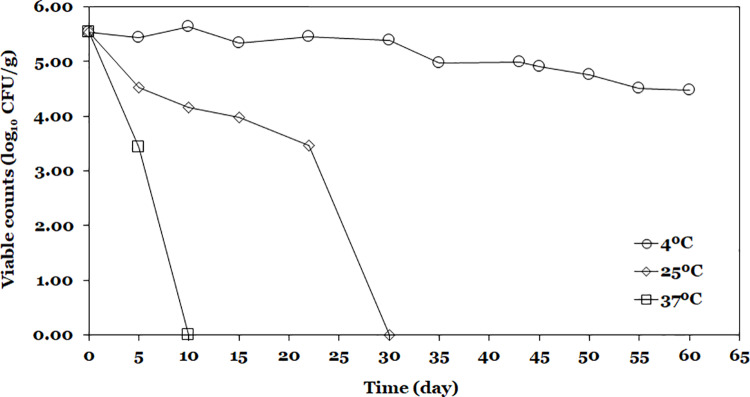
Evolution of viability of feed supplemented with SD probiotic powder during storage at three temperatures.

Finally, the viability of *L. lactis* A12 in fish feed is described in Fig 8. After 10 and 30 days, *L. lactis* A12 lost 100% of its viability at 37 and 25°C, respectively. During 30 days at 4 °C, *L. lactis* A12 was stable with a viable count of 5.39 log_10_ CFU/g of feed, with a survival rate of 97.46%. After this period, viable cell counts started to decrease to a final cell count of 4.47 ± 0.14 log_10_ CFU/g of feed resulting in a survival rate of 80.83% after 60 days of storage. Probiotic bacteria exhibited higher cellular viability loss rate (*k*_*m*_, day^-1^) at higher storage temperatures with values of -0.0149, -01462, and -0.5264 day^-1^ for 4, 25, and 37 °C, respectively. Using the *k*_*m*_ parameter is possible to predict the amount of probiotics that could be added to the fish feed to ensure the desired viability at the end of the shelf life of the product. Bacterial reductions for probiotic bacteria supplemented in fish feed in this study (0.14 log_10_) at 4 °C were lower than those reported by Melo-Bolívar et al. [50] who freeze-dried a mixture of the *L. lactis* A12 and *Priestia* species in BHI broth with fish feed reporting bacterial reductions between 0.44–0.57 log_10_. This supplemented feed (5.71 log_10_/g) was administered to fingerling of Nile Tilapia *in vivo* trials and improved growth performance, immune regulation, and resistance to *S. agalactiae* [86].

For both encapsulated probiotics [35,42] and supplemented feed [50] lower storage temperature exhibited higher survival rates. The use of wall materials as encapsulating agents improves stability during the storage due to their protective effect against environmental factors such as light and exposure to oxygen that could induce chemical reactions such as lipid oxidation that affect negatively probiotic viability [24]. Moreover, they assure an adequate physical state, providing a glassy matrix of low hygroscopicity which minimizes chemical reactions due to kinetic limitations as well as water uptake. However, the higher employed temperatures affected bacteria viability probably due to the oxidation of membrane lipids and denaturation of proteins that lead to the degradation of macromolecules in bacterial cells [110]. Even though the selected wall materials assure an adequate physical state. They could interact among them and offer wide possibilities to interact with the labile encapsulated agent. Also, the complexity of microorganisms difficult to achieve the desired protection for all the conditions assessed in the present work.

## 3. Conclusion

It was possible to establish a methodology to produce a spray-dried powder of probiotic *L. lactis* A12 intended for fish nutrition using a double purpose agro-industrial by-products – based culture media. The atomization pressure and the wall material mixture play significant roles in influencing probiotic performance such as its tolerance to acidic and bile salt environments. High air pressure and a high concentration of whey in the feed have been shown to improve the survival of *L. lactis* A12 after processes such as drying, exposure to low pH, or bile salts. The incorporation of wall materials not only enhances the physical and thermal properties of spray-dried powder but also contributes to the stability of probiotic bacteria during storage at 4 °C. It can be suggested that water activity and moisture content were not related to decreasing bacterial count during storage at 25 and 37 °C. Furthermore, feed supplemented with probiotic bacteria demonstrates good stability under refrigeration conditions. These findings indicate that both the stand-alone probiotic and its incorporation in fish feed could potentially be utilized in *in vivo* trials to validate *in vitro* findings and explore the potential health benefits in fish.
